# A Fully Unsupervised Deep Learning Framework for Non-Rigid Fundus Image Registration

**DOI:** 10.3390/bioengineering9080369

**Published:** 2022-08-05

**Authors:** Giovana A. Benvenuto, Marilaine Colnago, Maurício A. Dias, Rogério G. Negri, Erivaldo A. Silva, Wallace Casaca

**Affiliations:** 1 Faculty of Science and Technology (FCT), São Paulo State University (UNESP), Presidente Prudente 19060-900, Brazil; 2 Institute of Mathematics and Computer Science (ICMC), São Paulo University (USP), São Carlos 13566-590, Brazil; 3 Science and Technology Institute (ICT), São Paulo State University (UNESP), São José dos Campos 12224-300, Brazil; 4 Institute of Biosciences, Letters and Exact Sciences (IBILCE), São Paulo State University (UNESP), São José do Rio Preto 15054-000, Brazil

**Keywords:** fundus image, image registration, deep learning, computer vision applications

## Abstract

In ophthalmology, the registration problem consists of finding a geometric transformation that aligns a pair of images, supporting eye-care specialists who need to record and compare images of the same patient. Considering the registration methods for handling eye fundus images, the literature offers only a limited number of proposals based on deep learning (DL), whose implementations use the supervised learning paradigm to train a model. Additionally, ensuring high-quality registrations while still being flexible enough to tackle a broad range of fundus images is another drawback faced by most existing methods in the literature. Therefore, in this paper, we address the above-mentioned issues by introducing a new DL-based framework for eye fundus registration. Our methodology combines a U-shaped fully convolutional neural network with a spatial transformation learning scheme, where a reference-free similarity metric allows the registration without assuming any pre-annotated or artificially created data. Once trained, the model is able to accurately align pairs of images captured under several conditions, which include the presence of anatomical differences and low-quality photographs. Compared to other registration methods, our approach achieves better registration outcomes by just passing as input the desired pair of fundus images.

## 1. Introduction

In ophthalmology, computing technologies such as computer-assisted systems and content-based image analysis are indispensable tools to obtain more accurate diagnoses and detect signals of diseases. As a potential application, we can cite the progressive monitoring of eye disorders, such as glaucoma [1] and diabetic retinopathy [2], which can be conveniently performed by inspecting retina fundus images [3]. In fact, in follow-up examinations conducted by eye specialists, a particularly relevant task is image registration [4,5], where the goal is to assess the level of agreement between two or more fundus photographs captured at different instants or even by distinct acquisition instruments. In this kind of application, issues related to eye fundus scanning, such as variations in lighting, scale, angulation, and positioning, are properly handled and fixed when registering the images.

In more technical terms, given a pair of fundus images, $I_{Mov}$ and $I_{Ref}$, the registration problem comprises determining a geometric transformation that best aligns these images and maximizing their overlap areas while facilitating the visual comparison between them. As manually verifying with the naked eye possible changes between two or more fundus photographs is arduous and error-prone, there is a necessity to automate such a procedure [6,7]. Moreover, the difficulty in comparing large fundus datasets by a human expert and the time spent by ophthalmologists to accomplish manual inspections are commonly encountered challenges in the medical environment.

In recent years, machine and deep learning (DL) have paved their way into image registration and other related applications, such as computer-aided diagnosis [8,9], achieving very accurate and stable solutions. However, despite the existence of several proposals in the image registration literature, Litjens et al. [10], and Haskins et al. [11] recently indicated that there is a lack of consensus on a categorical technique that benefits from the robustness of deep learning towards providing high-accuracy registrations regardless of the condition of the acquired image pair. In addition, among methods specifically developed to cope with eye fundus registration, there is only a limited number of proposals that apply DL strategies, and most of them are focused on the supervised learning paradigm, i.e., the methods usually assume ground-truth reference data to train an alignment model. As reference data can be automatically generated by specific techniques or acquired through manual notes by an eye professional, both cases may suffer from the following drawbacks: (a) synthetically generating benchmark data can affect the accuracy of the trained models [12], and (b) manually annotating data are prone to failure due to the high number of samples to be labeled by a human agent, which includes the complication of creating full databases, large and representative enough in terms of ground-truth samples to be used to train a DL model effectively [11,13]. Lastly, dealing with ethical issues is another difficulty imposed when one tries to collect a large database of labeled medical images.

Aiming to address most of the issues and drawbacks raised above, in this paper, we propose a new methodology that combines two DL-based architectures into a fully unsupervised approach for retina fundus registration. More specifically, a U-shaped fully convolutional neural network (CNN) [14] and a spatial-transformer-type network [15] are integrated, so that the former produces a set of matching points from the fundus images, while the latter utilizes the mapped points to obtain a correspondence field used to drive geometric bilinear interpolation. Our learning scheme takes advantage of a benchmark-free similarity metric that gauges the difference between fixed and moving images, allowing for the registration without taking any prelabeled data to train a model or a specific technique to synthetically create training data. Once the integrated methodology is fully trained, it can achieve one-shot registrations by just passing the desired pair of fundus images.

A preliminary study of our learning scheme appears in our recently published ICASSP paper [16]. Going beyond our previous investigation, several enhancements are put forward. First, we extend our integrated DL framework to achieve more accurate outcomes, leading to a more assertive and stable registration model. We also provide a comprehensive literature review classifying popular and recent DL-based registration methods according to their network types, geometric transformations, and the general category of medical images (see Section 2). An extensive battery of new experiments and assessments are now given, in particular, the analysis of two additional fundus databases, the inclusion of new registration methods in the comparisons, and an ablation study covering the refinement step of our registration framework (see Section 3). Lastly, we also show that our learning registration pipeline can succeed with multiple classes of eye fundus images (see Section 4), a trait hard to be found in other fundus image registration methods.

In summary, the main contributions introduced by our approach are:

- A fully automatic learning strategy that unifies a context-aware CNN, a spatial transformation network and a label-free similarity metric to perform fundus image registration in one-shot without the need for any ground-truth data.
- Once trained, the registration model is capable of aligning fundus images of several classes and databases (e.g., super-resolution, retinal mosaics, and photographs containing anatomical differences).
- The combination of multiple DL networks with image analysis techniques, such as isotropic undecimated wavelet transform and connected component analysis, allowing for the registration of fundus photographs even with low-quality segments and abrupt changes.

## 2. Related Work

The literature covers a large number of DL-driven applications for clinical diagnosis in ophthalmology. Recently, several studies have been conducted on deep learning for the early detection of diseases and eye disorders, which include diabetic retinopathy detection [17,18], glaucoma diagnosis [19,20], and the automated identification of myopia using eye fundus images [21]. All these DL-based applications have high clinical relevance and may prove effective in supporting the design of suitable protocols in ophthalmology. Going deeper into DL-based applications, the image translation problem has also appeared in different ophthalmology image domains, such as image super resolution [22], denoising of retinal optical coherence tomography (OCT) [23], and OCT segmentation [24]. For instance, Mahapatra et al. [22] introduced a generative adversarial network (GAN) to increase the resolution of fundus images in order to enable more precise image analysis. Aiming at solving the issue of image denoising in high- and low-noise domains for OCT images, Manakov et al. [23] developed a model on the basis of the cycleGAN network to learn a mapping between these domains. Still on image translation, Sanchez et al. [24] combined two CNNs, the Pix2Pix and a modified deep retinal understanding network, to achieve the segmentation of intraretinal and subretinal fluids, and hyper-reflective foci in OCT images. For a comprehensive survey of image translation applications, see [25].

We now focus on discussing particular approaches for solving the image registration task. We split the registration methods into two groups: those that do not use DL (traditional methods), and those that do. Since our work seeks to advance the DL literature, we focus our discussion on this particular branch.

Considering the general application of image registration in the medical field, the literature has recently explored DL as a key resolution paradigm, including new approaches to obtain highly accurate results for various medical image categories, as discussed by Litjens et al. [10], Haskings et al. [11], and Fu et al. [26]. Most of these approaches rely on supervised learning, requiring annotated data to train a model. For example, Yang et al. [27] introduced an encoder–decoder architecture to carry out the supervised registration of magnetic resonance images (MRI) of the brain. Cao et al. [28] covered the same class of images, but they employed a guided learning strategy instead. Eppenhof and Pluim [29] also applied a supervised approach, but for registering chest computed tomography (CT) images through a U-shaped encoder-decoder network [30]. Still concerning supervised learning, several works attempted to compensate for the lack of labeled data by integrating new metrics into an imaging network. Fan et al. [31] induced the generation of ground-truth information used to perform the registration of brain images. Hering et al. [32] utilized a weakly supervised approach to align cardiac MRI images, and Hu et al. [33] took two networks: the former applied an affine transformation, while the latter gave the final registration of patients with prostate cancer.

More recently, new registration methods were proposed to circumvent the necessity of annotated data when training neural networks [15,34,35,36,37,38]. Jun et al. [34] presented a registration method that relied on a spatial transformer network (STN) network and a resampler for inspiration or expiration images of abdominal MRI. Zhang [35] covered the specific case of brain imaging, implementing two fully convolutional networks (FCNs), one to predict the parameters of a deformable transformation to align the fixed image to the moving image, and the other to proceed with the opposite alignment from moving image to a fixed one. Kori et al. [36] proposed a method that focused on exploring specific features of multimodal images by using a pretrained CNN followed by a keypoint detector, while the framework designed by Wang et al. [37] learn a modality-independent representation from an architecture composed of five subnets: an encoder, two decoders, and two transformation networks. Still on the registration of nonretinal cases, the method developed by Vos et al. [15] aligned cardiac images by comparing similar pixels to optimize the parameters of a CNN applied during the learning process. The method presented by Balakrishnan et al. [38] is another example of nonretinal registration, where the authors took a spatial transformation and U-Shaped learning scheme to explore brain MR data.

Concerning the DL-based methods specifically designed to handle retinal fundus images, Mahapatra et al. [39] presented a generative adversarial network (GAN) to align fundus photographs formed by two networks, a generator and a discriminator. While the former maps data from one domain to the other, the latter is tasked with discerning between true data and the synthetic distribution created by the generator [11]. Wang et al. [40] introduced a framework composed of two pretrained networks that perform the segmentation, detection, and description of retina features. Recently, Rivas-Villar et al. [41] have proposed a feature-based supervised registration method for fundus images where a network is trained using reference points transformed into heat maps to learn how to predict these maps in the inference step. The predicted maps are converted back into point locations and then used by a RANSAC-based matching algorithm to create the transformation models. Despite their capability in specifically solving the fundus registration problem, the methods described above employ reference data to compose the loss function.

In summary, most registration methods rely on supervised learning or take synthetically generated data in order to be effective. While generating new labels can overcome the scarcity of reference data, it also introduces an additional complication in modeling the problem, raising the issue of the reliability of artificially induced data in the medical image domain [42]. Another common trait shared by most DL registration methods is that they only produce high-accuracy outputs for a certain class of medical images or even subcategories of fundus photographs, such as super-resolution and retinal mosaics.

Table 1 summarizes the main DL registration methods discussed above.

## 3. Materials and Methods

### 3.1. Overview of the Proposed Approach

**Figure 1:**
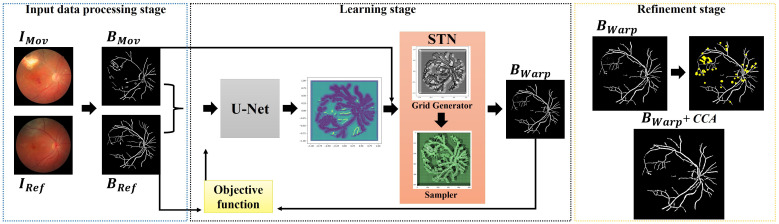
Overview of the proposed registration workflow.

The proposed framework seeks to align a pair of fundus images, $I_{Mov}$ and $I_{Ref}$, without the need for any labeled data. First, we extract the blood veins, bifurcations, and other relevant compositions of the eye, producing images $B_{Mov}$ and $B_{Ref}$ that are passed through a U-shaped fully convolutional neural network that outputs a correspondence grid between the images. In the next learning step, a matching grid is taken as input by a spatial transformation layer that computes the transformation model used to align the moving image. In our integrated architecture, the learning occurs through an objective function that measures the similarity between the reference and transformed images. As a result, the unified networks learn the registration task without the need for ground-truth annotations and reference data. Lastly, as a refinement step, we apply a mathematical morphology-based technique to remove noisy pixels that may appear during the learning process. Figure 1 shows the proposed registration approach.

### 3.2. Network Input Preparation

This step aims to handle the image pairs, $I_{Ref}$ and $I_{Mov}$, to improve the performance of the networks. In our approach, the images were resized to 512×512 to reduce the total number of network parameters related to the image sizes, thus leveraging the process of training the registration model. Next, a segmentation step was performed to obtain the eye’s structures that may be more relevant to the resolution of the registration problem. These include the blood vessels and the optic disc, as we can see from images $B_{Ref}$ and $B_{Mov}$ in the leftmost frame in Figure 1. To maximize the segmentation accuracy, we applied the isotropic undecimated wavelet transform (IUWT) [43] technique, which was developed specifically for the detection and measurement of retinal blood vessels.

### 3.3. Learning a Deep Correspondence Grid

**Figure 2:**
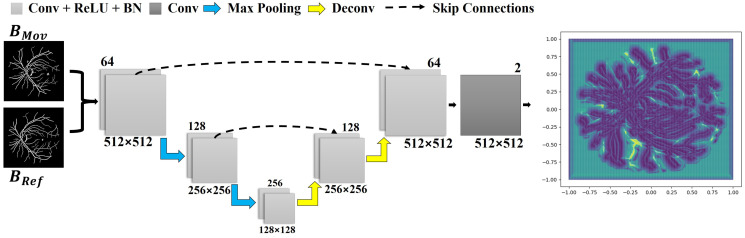
The implemented network architecture, used to obtain a correspondence grid. Each layer is represented by a block with a distinct color. Below each block, the data resolution is described, while in the upper-right corner, the number of kernels per layer is shown. The correspondence grid is the network’s output, as displayed in the rightmost corner.

As mentioned before, the first implemented learning mechanism assumes a U-Net-type structure whose goal is to compute a correspondence grid for the reference and moving images. The network input is formed by the pair $B_{ref}$ and $B_{Mov}$, which is passed through the first block of convolutional layers. This network comprises two downsample blocks: a max pooling layer and two convolution layers, as illustrated in Figure 2. In each block, the size of the input is decremented in half according to the resolution of the images, while the total number of analyzed features doubles.

In the second stage, two blocks are added as part of the network upsampling process. These are composed of a deconvolution layer, which accounts for increasing the input size while decreasing the number of features processed by the network, and two convolutional layers. The resultant data from the deconvolution are then concatenated with the data obtained by the output of the convolution block at the same level from the previous step (see the dashed arrows in Figure 2). In our implementation, the ReLU activation function and a batch normalization layer were used in each convolutional layer except for the last one. The last convolutional layer enables to return a correspondence field compatible with the dimension of the input data.

The network outputs a grid of points (i.e., the correspondence grid), which is used to drive the movement of each pixel when aligning the pair of images. The rightmost quiver plot in Figure 2 displays the correspondence grid, where the arrows moved from the coordinates of the regular grid to the positions produced by the network, while the purple and yellow maps show the points of highest and lowest mobility, respectively.

### 3.4. Learning a Spatial Transformation

In this step, we took an adaptation of the spatial transformer network architecture [44] to obtain a transformation model for mapping $B_{Mov}$. Particularly, the STN structure allows for the network to dynamically apply scaling, rotation, slicing, and nonrigid transformations on the moving image or feature map without the requirement for any additional training supervision or lateral optimization process.

The STN network incorporated as part of our integrated learning scheme consists of two core modules: grid generator and sampler. The goal of the grid generator is to iterate over the matching points previously determined by the U-shaped network to align the correspondence positions in target image $B_{Mov}$. Once the matches are properly found, the sampler module extracts the pixel values at each position through a bilinear interpolation, thus generating the definitive transformed image $B_{Warp}$. Figure 1 (middle frame) illustrates the implemented modules of STN.

### 3.5. Objective Function

Since registration is performed without using any set of labeled data, the objective function used to train our approach consists of an independent metric that gauged the similarity degree between the images. In more mathematical terms, we took the normalized cross-correlation (NCC) as a measure of similarity for the objective function:

(1) $$NCC\left(x,y\right)\;=\;\frac{\sum_{i=0}^{m}\sum_{j=0}^{n}T_{i,j}R_{i,j}}{\sqrt{\;\left(\sum_{i=0}^{m}\sum_{j=0}^{n}T_{i,j}^{2}\;\right)\;\;\left(\sum_{i=0}^{m}\;\sum_{j=0}^{n}R_{i,j}^{2}\;\right)\;\;}}\;.$$

In Equation (1), $T_{i,j}=t\left(x+i,y+j\right)-{\bar{t}}_{x,y}$, $R_{i,j}=r\left(i,j\right)-\bar{r}$, and t(i,j) and r(i,j) are the pixel values at (i,j) regarding the warped and reference images, $B_{Warp}$ and $B_{Ref}$, respectively, while $\bar{r}$ and $\bar{t}$ give the average pixel values w.r.t. $B_{Ref}$ and $B_{Warp}$ [45]. In Equation (1) the objective (fitness) function is maximized, as the higher the NCC is, the more similar (correlated) the two images are.

The NCC metric can also be defined in terms of a dot product where the output is equivalent to the cosine of the angle between the two normalized pixel intensity vectors. This correlation allows for standard statistical analysis to ascertain the agreement between two datasets, which is frequently chosen as a similarity measure due to its robustness [46], high-accuracy and adaptability [47].

### 3.6. Refinement Process

Since our approach allows for nonrigid registrations, transformed image $B_{Warp}$ may hold some noisy pixels, especially for cases where the images to be aligned are very different from each other. In order to overcome this, we applied a mathematical morphology technique called connected component analysis (CCA) [48].

CCA consists of creating collections of objects formed by groups of adjacent pixels of similar intensities. As a result, eye fundus structures are represented in terms of their morphologically continuous structures, such as connected blood vessels. We, therefore, can identify and filter out small clusters of noisy pixels (see the yellow points in the rightmost frame in Figure 1) from a computed set of connected morphological components.

### 3.7. Datasets and Assessment Metrics

In order to assess the performance of the registration methodology, we took three retina fundus databases. The specification of each data collection is described below.

- **FIRE**—A full database containing several classes of high-resolution fundus images, as detailed in [49]. This data collection comprises 134 pairs of images, grouped into three categories: A, S, and P. Categories A and S covers 14 and 71 pairs of images, respectively, whose fundus photographs present an estimated overlap of more than 75%. Category A also includes images with anatomical differences. Category P, on the other hand, is formed by image pairs with less than 75% of estimated overlap.
- **Image Quality Assessment Dataset (Dataset 1)**—this public dataset [50] is composed of 18 pairs of images captured from 18 individuals, where each pair is formed by a poor-quality image (blurred and/or with dark lighting with occlusions), and a high-quality image of the same eye. There are also pairs containing small displacements caused by eye movements during the acquisition process.
- **Preventive Eye Exams Dataset: (Dataset 2)**—a full database containing 85 pairs of retinal images provided by an ophthalmologist [7]. This data collection gathers real cases of acquisitions such as monitoring diseases, the presence of artifacts, noise, and excessive rotations, i.e., several particular situations typically faced by ophthalmologists and other eye specialists in their routine examinations with real patients.

Aiming at quantitatively assessing the registration results, four validation metrics were adopted: mean squared error (MSE) [36,39], structural similarity index measure (SSIM) [36], Dice coefficient (Dice) [15,28,31,37,40,51] and gain coefficient (GC) [7,52].

The MSE is a popular risk metric that computes the squared error between expected and real values, as shown in Equation (2):

(2) $$MSE\left(B_{Ref},B_{Warp}\right)=\frac{1}{H\times W}\sum_{x=0}^{W}\sum_{y=0}^{H}{\left(B_{Ref_{\left(x,y\right)}}-B_{Warp_{\left(x,y\right)}}\right)}^{2}\;,$$

where *H* and *W* represent the dimensions of the images $B_{Ref}$ and $B_{Warp}$. The values of the MSE range from 0 to infinite. The closer MSE is to zero, the better.

The SSIM metric takes the spatial positions of the image pixels to calculate the so-called similarity score, as determined by Equation (3):

(3) $$SSIM\left(B_{Ref},B_{Warp}\right)=\frac{\left(2\mu_{B_{Ref}}\mu_{B_{Warp}}+c_{1}\right)\left(2\sigma_{B_{Ref}B_{Warp}}+c_{2}\right)}{\left(\mu_{B_{Ref}}^{2}+\mu_{B_{Warp}}^{2}+c_{1}\right)\left(\sigma_{B_{Ref}}^{2}+\sigma_{B_{Warp}}^{2}+c_{2}\right)}\;.$$

In Equation (3), μ represents the mean value of the image pixels, σ is the variance, $\sigma^{2}$ gives the covariance of $B_{Ref}$ and $B_{Warp}$, and $c_{1}$ and $c_{2}$ are variables used to stabilize the denominators. The results are concentrated into a normalized range of 0 and 1, with 0 being the lowest score for the metric, and 1 the highest.

The Dice coefficient is another metric extensively used in the context of image registration, which varies between 0 and 1, where 1 indicates an overlap of 100% . Equation (4) rules the mathematical calculations of this metric:

(4) $$Dice\left(B_{Ref},B_{Warp}\right)=\frac{2\times B_{Ref}\cap B_{Warp}}{B_{Ref}\cup B_{Warp}}\;.$$

The GC metric, as described by Equation (5), compares the overlap between the images $B_{Ref}$ and $B_{Warp}$, and the pair of images $B_{Ref}$ and $B_{Mov}$ [52]. Thus, if the number of pixels aligned after the transformation is equal to the number of pixels before the image is transformed, the result is equal to 1. The more pixels are aligned compared to the original overlap, the greater the overlapping value.

(5) $$GC\left(B_{Ref},B_{Mov},B_{Warp}\right)=\frac{|B_{Ref}\cap B_{Warp}|}{|B_{Ref}\cap B_{Mov}|}\;.$$

### 3.8. Implementation Details and Training

Our computational prototype was implemented using Python language with the support of libraries for image processing and artificial intelligence routines such as OpenCV [53], Scikit-learn [54] and Tensorflow [55].

**Figure 3:**
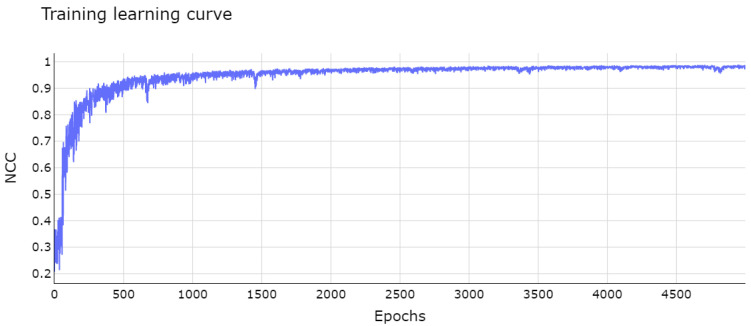
Network learning curve after 5000 epochs. The vertical axis represents the fitness value, which is maximized during training, for each epoch on horizontal axis.

The module of integrated networks was trained with batches of eight pairs of images for 5000 epochs. The plot in Figure 3 shows the learning curve of the integrated networks. The curve exponentially increased with a few small oscillations, converging in the first 2000 epochs and remaining stable towards the end of this phase. The learning process was optimized with the ADAM algorithm [56], a mathematical method based on the popular stochastic descending gradient algorithm. The training was performed on a cluster with 32GB of RAM and two Intel(R) Xeon(R) E5-2690 processors.

**Figure 4:**
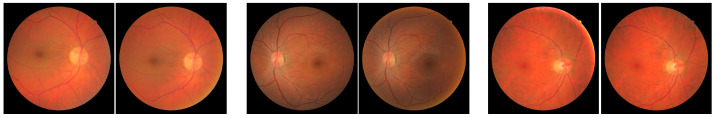
Fundus image pairs typically used for training.

The images used in the training step were taken from the category S testing set of the FIRE database, which gathers fundus images of 512×512 pixels. This particular category was chosen for training because it comprised the largest and most comprehensive collection of images in the FIRE database, covering pairs of retina images that are more similar to each other (see Figure 4 for an illustrative example). An exhaustive battery of tests showed that this full dataset is effective for training, as the conducted tests revealed that the presence of images with low overlapping levels avoids oscillations in the learning curve of the network, leading to a smaller number of epochs for convergence.

Another observable aspect when using our approach is that the registration model was trained by taking a moderately sized dataset of fundus images—a trait that can also be found in other fundus photography related applications, such as landmark detection [41] and even for general applications of DL-type networks [57].

## 4. Results and Discussion

In this section, we present an ablation study concerning the refinement stage of our methodology, which includes the analysis of different settings to increase the quality of the registration results. We also provide and discuss a comprehensive experimental evaluation of the performance of our approach by comparing it with recent image registration methods from both quantitative as well as qualitative aspects.

### 4.1. Ablation Study

We start by investigating whether the CCA technique can be applied to improve the registration results. We thus incorporated CCA as part of our framework, verifying its impact quantitatively and visually. We compared the application of such a technique by taking three distinct threshold values used to discard clusters with noisy pixels. We also compared the submodels derived from CCA + registration networks against two popular digital image processing techniques: opening and closing morphological filters.

**Figure 5:**
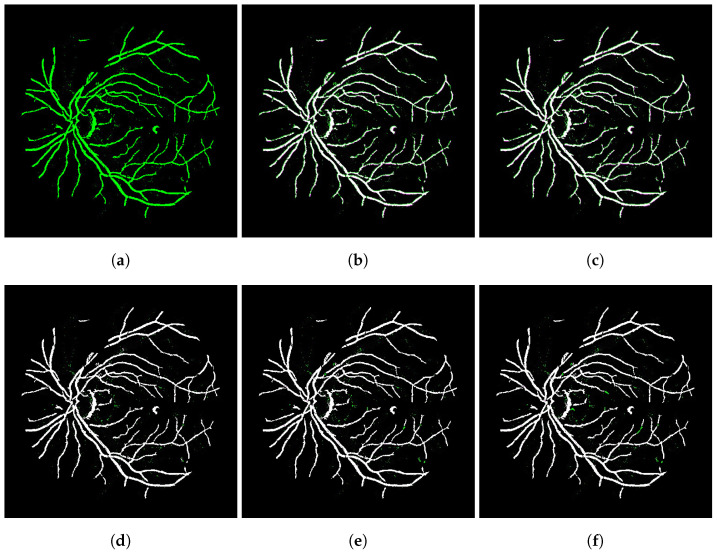
Visual comparison for several denoising strategies applied on transformed images generated by the integrated networks. (**a**) Network—SSIM: 0.9338; (**b**) Opening—SSIM: 0.8640; (**c**) Closing—SSIM: 0.8625; (**d**) CCA 10—SSIM: 0.9613; (**e**) CCA 20—SSIM: 0.9611; (**f**) CCA 30—SSIM: 0.9598.

Table 2 lists the average of the evaluation metrics for each submodel and database. The standard deviation is also tabulated in parentheses. By verifying the scores achieved by the morphological transformations (network + opening and network + closing), one can conclude that they did not lead to an improvement in quality for the registered image pairs, even for those containing noise. Moreover, the application of these morphology-based filters may alter the contour of the structures present in the images, as shown in Figure 5a,c.

On the other hand, by comparing the results output by submodels network + CCA, we noticed that they clearly contributed to a substantial gain in registration quality in all examined datasets, as one can see from the scores highlighted in bold in Table 2.

In Figure 5, the image registered by the integrated networks without any refinement process appears in green (Figure 5a), while the others are comparisons between these and the images after applying each denoising technique, and they assume a magenta color so that when added to the green image lead to white pixels. In this way, the noise data in green indicate the pixels that were treated in these images. Visually speaking, when comparing the results in Figure 5e,f, the noise was substantially reduced after applying the CCA technique.

From the conducted ablation analysis, we included as part of our full registration framework the application of CCA algorithm with a threshold value of 20 pixels.

### 4.2. Comparison with Image Registration Methods

We compare the outputs obtained by our approach against the ones produced by four modern image registration methods. Within the scope of keypoint-based techniques, the algorithms proposed by Wang et al. [58] and Motta et al. [7], called GFEMR and VOTUS, were considered in our analysis. For comparisons covering DL-based methods, we ran the techniques proposed by Vos et al. [59], DIRNet, and the weakly supervised strategy introduced by Hu et al. [33]. These DL-driven algorithms were tuned following the same experimental process performed by our approach, i.e., they were fully trained with the same group of training samples, taking into account the same amount of epochs.

**Figure 6:**
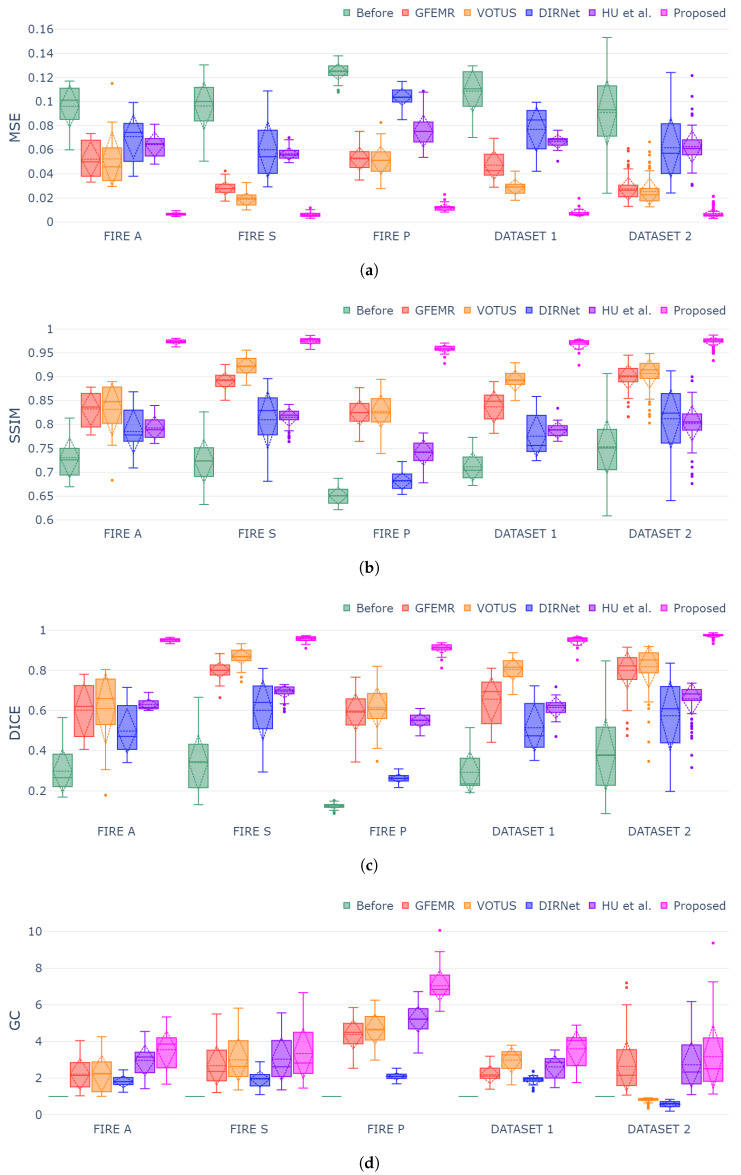
Box-plot charts for each evaluation metric and dataset. Symbols (↓) and (↑) indicate that “lower is better” and “higher is better”, respectively. (**a**) Box-plot distribution for MSE metric (↓); (**b**) box-plot distribution for SSIM metric (↑); (**c**) box-plot distribution for Dice metric (↑); (**d**) box-plot distribution for GC metric (↑).

Figure 6a–d show box plots for each validation metric and registration dataset. The generated plots show that the proposed framework outperformed both conventional and DL-based techniques in all instances, demonstrating consistency and stability for different categories of fundus images. The MSE, SSIM and Dice metrics exhibited similar behavior while still holding the smallest variation in the box plots, thus attesting to the capability of our approach in achieving high-accuracy registrations regardless of the pair of fundus images. Lastly, concerning the GC metric (Figure 6d), since such a measure gauges the overlap segments before and after the registration, the datasets holding more discrepant images were the ones that produced higher scores, as one can check for Category P of FIRE database. DIRNet and VOTUS remain competitive for Category S of FIRE, but they were still outperformed by the proposed methodology. A similar outcome was found when DIRNet was compared to our approach for Dataset 2.

A two-sided Wilcoxon test at 5% significance level was applied to verify the statistical validity of the registrations produced by our approach against the ones delivered by other methods. From the *p*-values in Table 3, the results from our approach were statistically more accurate than others in all datasets for at least three of the four evaluation metrics (MSE, SSIM and DICE). Moreover, we can check that our approach was statically superior (p<0.05) in 96 of the 100 tests conducted, thus attesting to the statistical validation of the obtained results.

**Figure 7:**
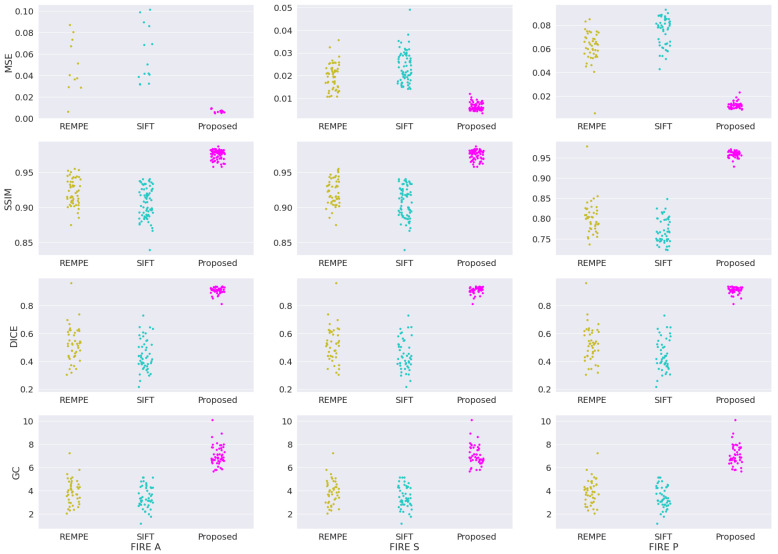
Sample distribution analysis for REMPE, SIFT, and our framework for the FIRE datasets.

In addition to the four registration methods already assessed in our validation study, we provide new assessments involving two new methods: the recent registration through eye modelling and pose estimation (REMPE) technique [60], and the well-established scale-invariant feature transform (SIFT) algorithm [61]. Figure 7 shows the box-plot distribution for each validation metric applied to categories A, S and P from FIRE database. The plotted box plot shows that our framework outperformed the REMPE and SIFT methods, achieving the smallest variations between outputs, which are visually represented by the tightest clusters in each plot.

**Figure 8:**
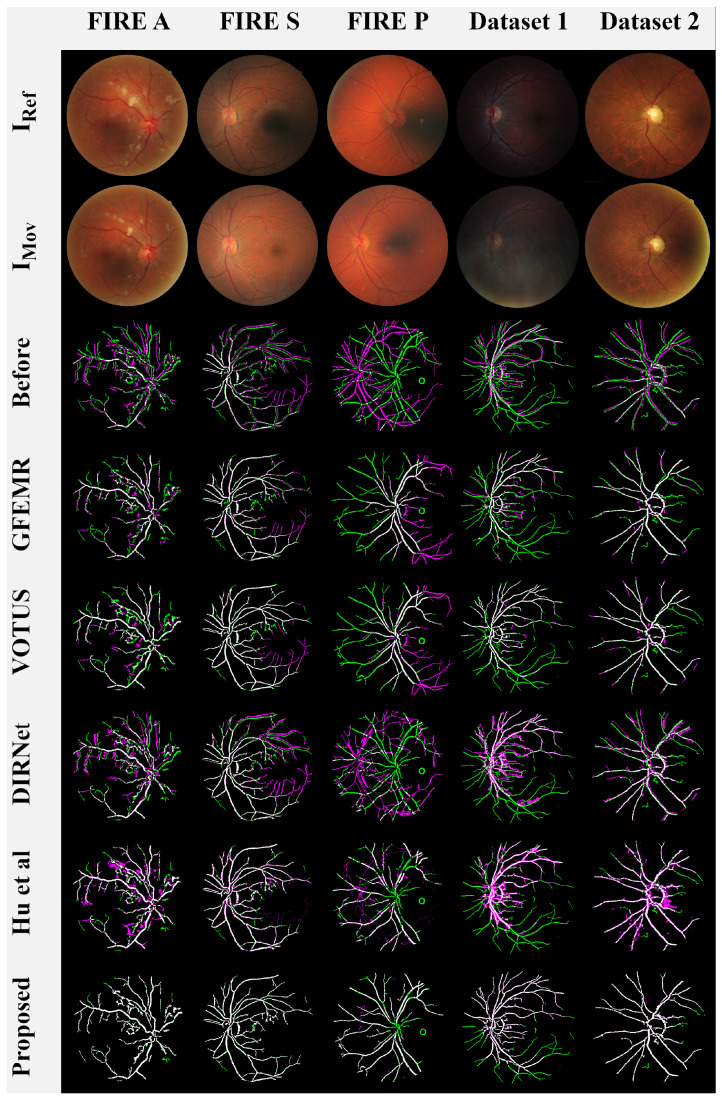
Visual analysis of the results. Lines 1 and 2: original images from each examined database, Line 3: the images before the registration process, Lines 4-9: the overlapping areas between $B_{Ref}$ (in green) and $B_{Warp}$ (in magenta) produced by each registration method.

A visual qualitative analysis of the registrations produced by the competing methods is presented in Figure 8. Here, we followed [7,16,52] to represent the aligned images in terms of color compositions to increase the visual readability and interpretation of the results. More specifically, images $B_{Ref}$ and $B_{Warp}$ were rendered in green and magenta, while the overlap of both images is in white, giving the level of agreement between them.

Keypoint-based approaches GEEMR and VOTUS produced acceptable results for most image pairs, but they are not yet able to satisfactorily deal with the blood veins located farther away from the eye globe. DL-based methods DIRNET and Hu et al. performed nonrigid registrations, causing deformations in the output images (e.g., see the misalignment and distortions in the first, third, and fourth images from Figure 8). Our framework also performs nonrigid registration; however, the implemented networks ensure that the transformation applied to moving image $B_{Mov}$ uniformly distorts the image structures, rendering $B_{Mov}$ closer to the reference image $B_{Ref}$. Lastly, one can verify that our registration model and that of Hu et al. were the ones that were capable of aligning the very hard images from Category P of the FIRE database.

Another relevant observation when inspecting Figure 8 is the role of vessels in our framework. Indeed, such a procedure allows for the method to carry out the registration under the most diverse conditions. For instance, the fundus images from Dataset 1 are composed of dark lighting, blur, and smoky occlusions. By handling the eye’s vessels, it is possible to highlight the vascular structure of these images, accurately performing the registration while avoiding the need for new exams to replace poorly captured photographs.

## 5. Conclusions

This paper introduced an end-to-end methodology for fundus image registration using unsupervised deep learning networks and morphological filtering. As shown by the conducted experiments, our approach was able to operate in a fully unsupervised fashion, requiring no prelabeled data or side computational strategy to induce the creation of synthetic data for training. After being trained, the current model produced one-shot registrations by just inputting a pair of fundus images.

From the battery of conducted experiments, it was verified that the proposed methodology produced very stable and accurate registrations for five representative datasets of fundus images, most of them covering several challenging cases, such as images with anatomical differences and very low-quality acquisitions. Furthermore, the methodology performed better than several modern existing registration methods in terms of the accuracy, stability, and capability of generalization for several datasets of fundus photographs. Visual representations of the registration results also revealed a better adherence achieved by the introduced framework in comparison with keypoint-based and DL-based methods.

As future work, we plan to: (i) analyze the effects of applying other fitness functions beyond NCC; (ii) investigate the use of other DL neural networks, for example, SegNet, X-Net and adversarial networks; and (iii) extend our framework to cope with specific clinical problems, including its adaptation for domain transformation, from fundus images to ultra-wide-field fundus photography [25], and 3D stereoscopic reconstruction of retinal images, which is another application related to the context of diagnostic assistance.

## Figures and Tables

**Table 1:** Survey of DL studies. Blue lines refer to works that specifically cover fundus registration.

Papers	Ref.	Images Type	Network	Architecture	Transformation
Yang et al.	[27]	Brain MRI (3D)	Supervised	Encoder + Decoder	Affine + Nonrigid (LDDMM)
Cao et al.	[28]	Brain MRI (3D)	Supervised	Network preparation + network learning	Affine + Nonrigid (TPS)
Eppenhof and Pluim	[29]	Chest CT (3D)	Supervised	Adapted U-Net	Nonrigid (B-Spline)
Fan et al.	[31]	Brain MRI (3D)	Weakly supervised	BIRNet	Nonrigid
Hering et al.	[32]	Cardiac MRI (3D)	Weakly supervised	Adapted U-Net	Nonrigid (B-Spline)
Hu et al.	[33]	TRUS and prostate MRI (3D)	Weakly supervised	Global Net + Local Net	Affine + Non-rigid
Mahapatra et al.	[39]	Retinal FA images + cardiac MRI (2D)	Weakly supervised	GAN	Nonrigid
Wang et al.	[40]	Multimodal retinal image	Weakly supervised	Segmentation network + feature detection and description network + outlier rejection network	Affine
Rivas-Villar et al.	[41]	Color fundus images	Weakly supervised	U-Net + RANSAC	Similarity transformation
Jun et al.	[34]	Abdominal MRI (2D and 3D)	Unsupervised	CNN + STN	Nonrigid (B-Spline)
Zhang	[35]	Brain MRI (3D)	Unsupervised	Adapted U-Net + 2 FCN	Nonrigid (B-Spline)
Vos et al.	[15]	Cardiac MRI and chest CT (3D)	Unsupervised	CNN Affine + CNN nonrigid	Affine + Nonrigid (B-Spline)
Wang et al.	[37]	Brain MRI (2D and 3D)	Unsupervised	Encoder + decoders + transformation networks	Affine + Nonrigid
Kori et al.	[36]	Brain MRI (3D)	Unsupervised	VGG-19 + transformation estimator	Affine
Balakrishnan et al.	[38]	Brain MRI (3D)	Unsupervised	Adapted U-Net + STN (+ information optional auxiliary)	Nonrigid (linear)

**Table 2:** Comparison of registration submodels created as variations of our framework. Values in bold indicate the best scores, and values in italics the second best.

Metrics	Methods	FIRE A	FIRE S	FIRE P	Dataset 1	Dataset 2
**MSE (↓)**	Network	0.0080 (0.0017)	0.0074 (0.0019)	0.0143 (0.0026)	0.0095 (0.0034)	0.0093 (0.0039)
	Network + Opening	0.0287 (0.0030)	0.0319 (0.0023)	0.0343 (0.0031)	0.0324 (0.0037)	0.0268 (0.0035)
	Network + Closing	0.0284 (0.0029)	0.0316 (0.0023)	0.0337 (0.0030)	0.0321 (0.0035)	0.0265 (0.0034)
	Network + CCA 10	*0.0068 (0.0015)*	**0.0062 (0.0017)**	*0.0121 (0.0027)*	**0.0079 (0.0034)**	**0.0071 (0.0038)**
	Network + CCA 20	**0.0068 (0.0014)**	**0.0062 (0.0017)**	**0.0120 (0.0027)**	*0.0079 (0.0035)*	**0.0071 (0.0038)**
	Network + CCA 30	0.0069 (0.0015)	0.0063 (0.0017)	*0.0121 (0.0027)*	0.0080 (0.0035)	**0.0071 (0.0038)**
**SSIM (↑)**	Network	0.9586 (0.0086)	0.9638 (0.0104)	0.9290 (0.0080)	0.9539 (0.0130)	0.9572 (0.0162)
	Network + Opening	0.8928 (0.0110)	0.8807 (0.0094)	0.8773 (0.0107)	0.8797 (0.0130)	0.9001 (0.0118)
	Network + Closing	0.8923 (0.0103)	0.8818 (0.0092)	0.8752 (0.0104)	0.8800 (0.0124)	0.8998 (0.0119)
	Network + CCA 10	*0.9731 (0.0055)*	**0.9749 (0.0068)**	0.9575 (0.0076)	**0.9682 (0.0128)**	*0.9733 (0.0106)*
	Network + CCA 20	**0.9732 (0.0053)**	*0.9748 (0.0068)*	**0.9585 (0.0075)**	*0.9681 (0.0133)*	**0.9734 (0.0103)**
	Network + CCA 30	0.9727 (0.0054)	0.9744 (0.0068)	*0.9580 (0.0073)*	0.9678 (0.0133)	*0.9733 (0.0102)*
**Dice (↑)**	Network	0.9399 (0.0121)	0.9484 (0.0143)	0.8915 (0.0237)	0.9363 (0.0268)	0.9295 (0.0425)
	Network + Opening	0.7814 (0.0101)	0.7743 (0.0121)	0.7367 (0.0173)	0.7807 (0.0359)	0.8046 (0.0382)
	Network + Closing	0.7874 (0.0090)	0.7798 (0.0117)	0.7465 (0.0171)	0.7860 (0.0331)	0.8086 (0.0369)
	Network + CCA 10	*0.9502 (0.0100)*	*0.9579 (0.0120)*	*0.9103 (0.0238)*	*0.9476 (0.0265)*	*0.9466 (0.0404)*
	Network + CCA 20	**0.9505 (0.0097)**	**0.9580 (0.0122)**	**0.9109 (0.0238)**	**0.9477 (0.0270)**	**0.9467 (0.0404)**
	Network + CCA 30	0.9496 (0.0100)	0.9573 (0.0123)	0.9097 (0.0236)	0.9471 (0.0270)	0.9463 (0.0404)
**GC (↑)**	Network	3.4237 (0.9921)	3.2125 (1.3424)	6.7499 (0.8029)	3.4786 (0.9630)	3.0494 (1.6853)
	Network + Opening	2.8025 (0.8065)	2.5910 (1.0920)	5.4621 (0.6265)	2.8544 (0.7680)	2.6075 (1.4265)
	Network + Closing	2.8733 (0.8394)	2.6515 (1.1326)	5.6395 (0.6508)	2.9203 (0.7960)	2.6565 (1.4714)
	Network + CCA 10	*3.5511 (1.0343 )*	**3.3379 (1.3973)**	**7.0506 (0.8443)**	**3.5963 (0.9943)**	**3.1755 (1.7625)**
	Network + CCA 20	**3.5520 (1.0361)**	*3.3378 (1.3965)*	*7.0410 (0.8410)*	*3.5956 (0.9940)*	*3.1716 (1.7571)*
	Network + CCA 30	3.5443 (1.0345)	3.3321 (1.3920)	7.0160 (0.8373)	3.5892 (0.9888)	3.1672 (1.7517)

**Table 3:** *p*-values from two-sided Wilcoxon test at 5% significance level applied to compare the proposed approach against other registration methods.

Metric	Method	Fire A	FIRE S	FIRE P	Dataset 1	Dataset 2
MSE	Before	<$10^{-7}$	0.0	0.0	<$10^{-9}$	0.0
	GFEMR	<$10^{-7}$	0.0	0.0	<$10^{-9}$	0.0
	VOTUS	<$10^{-7}$	0.0	0.0	<$10^{-9}$	0.0
	DIRNet	<$10^{-7}$	0.0	0.0	<$10^{-9}$	0.0
	HU et al.	<$10^{-7}$	0.0	0.0	<$10^{-9}$	0.0
SSIM	Before	<$10^{-7}$	0.0	0.0	<$10^{-9}$	0.0
	GFEMR	<$10^{-7}$	0.0	0.0	<$10^{-9}$	0.0
	VOTUS	<$10^{-7}$	0.0	0.0	<$10^{-9}$	0.0
	DIRNet	<$10^{-7}$	0.0	0.0	<$10^{-9}$	0.0
	HU et al.	<$10^{-7}$	0.0	0.0	<$10^{-7}$	0.0
DICE	Before	<$10^{-7}$	0.0	0.0	<$10^{-9}$	0.0
	GFEMR	<$10^{-7}$	0.0	0.0	<$10^{-9}$	0.0
	VOTUS	<$10^{-7}$	0.0	0.0	<$10^{-9}$	0.0
	DIRNet	<$10^{-7}$	0.0	0.0	<$10^{-9}$	0.0
	HU et al.	<$10^{-7}$	0.0	0.0	<$10^{-9}$	0.0
GC	Before	<$10^{-7}$	0.0	0.0	<$10^{-9}$	0.0
	GFEMR	0.0017	0.0028	0.0	0.0001	0.0253
	VOTUS	0.0058	0.1206	0.0	0.0224	0.0
	DIRNet	0.0	0.0	0.0	0.0	0.0
	HU et al.	0.1139	0.1994	0.0	0.0037	0.1594

## Data Availability

The computational framework was implemented in Python language using libraries provided by OpenCV: https://opencv.org (accessed on 11 August 2021), Scikit-learn: https://scikit-learn.org/stable/ (accessed on 14 September 2021) and TensorFlow: https://www.tensorflow.org/ (accessed on 22 September 2021). The public databases cited in the Section 3.7 are freely available at: https://projects.ics.forth.gr/cvrl/fire/ (accessed on 15 July 2021) and https://www5.cs.fau.de/research/data/fundus-images/index.html (accessed on 15 July 2021).
